# Supplementary material for: The prevalence of metabolic conditions before and during the COVID-19 pandemic and its association with health and sociodemographic factors
Source: PLoS One. 2023 Feb 10;18(2):e0279442. doi: 10.1371/journal.pone.0279442 (PMC9916641; doi:10.1371/journal.pone.0279442)
Supplement: Attachment — Submitted filename: PONE-D-22-21818R2_R2R.docx [file pone.0279442.s003.docx]

**Reviewer #1:**

**Comment**

1. In the Abstract, suggest changing 'rationing' to 'allocation'.

**Response**
We have changed it as suggested.

**Comment**
2. In the Abstract, where it refers to ' older adults, non-Hispanic Black people... individuals with poor health status', no data are presented to support this statement in the Abstract. Either include supporting evidence in the Abstract or omit.

**Response**
We have deleted the emphasis as suggested.

**Reviewer #2:**

**Comment**

Thank you for addressing every point and incorporating them into your revised manuscript.
The paper sounds much better indeed with feedback of reviewer 1 as well.

**Response**

Thank you for helping to improve the paper substantively and stylistically.
