# Supplementary material for: The prevalence of metabolic conditions before and during the COVID-19 pandemic and its association with health and sociodemographic factors
Source: PLoS One. 2023 Feb 10;18(2):e0279442. doi: 10.1371/journal.pone.0279442 (PMC9916641; doi:10.1371/journal.pone.0279442)
Supplement: Attachment — Submitted filename: PLOS ONE-Reviewer comments.docx [file pone.0279442.s001.docx]

**Review of Manuscript Number: PONE-D-22-21818**

**Introduction:**

The introduction is lacking the part linking the risk factors especially mental health status during pandemic to cardiometabolic diseases and how COVID-19 pandemic has led to an increased adoption of such behavioral risk factors e.g., physical inactivity and smoking.

- **Line 62-64**

COVID-19 may have an exacerbating effect on glycemic control for patients with diabetes [14, 20], and there may be risk of increased body mass index (BMI) as well as a deterioration in glucose regulation due to COVID-19 [16, 21].

**Reviewer comments:**

Will you clarify if COVID-19 infection or the implications of COVID-19 related lockdown have led to exacerbation of glycemic control and increased BMI?

**Methods:**

- **Line 90-91**

Briefly, the 2019 survey (HINTS 5 Cycle 3) was conducted from January through April 2019, and the 2020 survey (HINTS 5 Cycle 4) was conducted from February through June 2020.

**Reviewer comments:**

COVID-19 was declared pandemic on March 11^th^, 2020, and lockdown has followed in most of the world regions. Therefore, if you are aiming to compare the rates and prevalence of cardiometabolic diseases, that are mainly linked to lifestyle behavioral risk factors taking time to accumulate and changing health conditions, before and during COVID-19, the results of this study might be biased and under-estimated.

- **Line 124-125**

The number of days per week of moderate intensity physical activity (none and at least one day per week)

**Reviewer comments:**

Why have you chosen to ask none or at least one day per week? Physical activity significance should hit the international recommendations of 150 mins/week. Therefore, performing one time/week or so will not add significant information, hence correlation. If you are not using a valid tool to measure your variables, you will be subjected to bias. It is better to question about the days and minuets and calculate the mean.

**Results**

**Line 147-151**

prevalence of diabetes was higher during the COVID-19 pandemic (18.10%) than before the 150 pandemic (17.28%). However, the prevalence of hypertension (36.36% vs. 36.38%) and obesity 151 (34.68% vs. 34.18%) was similar during and before the pandemic.

**Reviewer comments:**

Again, the slight variations in the prevalence of cardiometabolic diseases between before and during the pandemic is most likely due to early trials on lifestyle related behavior that work in cumulative effects manner (developing over longer period) developing chronic diseases.

- **Line 163-166**

Individuals who were former cigarette smokers or current smokers had increased prevalence of cardiometabolic conditions. For ecigarette use groups, the prevalence had increased for those who had never used e-cigarettes and those who currently used e-cigarettes however decreased for former e-cigarette users.

**Reviewer comments:**

The prevalence was increased for those who never smoked e-cigarettes and those who currently use e cigarette, is bringing so much confusion for the reader and later to decision makers. You need to revisit your analysis or at least explain your odd findings in the discussion section comparing to previous research.

**Discussion**

- **Line 200-202**

**Reviewer comments:**

Discussion needs to be improved.

1. Correlation of age and risk of cardiometabolic conditions is not well highlighted. First, your study revealed that age group from 26- ≥65 year are at increased risk when compared to 18-25 years. I can notice that the risk is doubled for 26-34 year and tripled for 35-49 year. Therefore, in your recommendations, you need to focus not only on older age group but middle-aged as well.
2. You need to enrich discussion further to add new implications of your results. Since the findings are not adding additional knowledge to the literature, you need to discuss your odd findings and give explanations. You may discuss how to improve future research in the same area e.g., by adopting validated tools to measure variables, by using more reliable source of data like registry or objective measures as compared to self-reported. Suggestions in how to improve the internal validity of the results are important and show your understanding of pitfalls. Therefore, add a section of “implications to practice & research’.

**Limitations:**

Kindly add the limitations discussed above and the probable effect of confounders not considered in this study such as sedentary behavior, sleep, eating patterns, employment and etc., that might give rise to inaccurate estimates of the true association.

Overall, the manuscript needs improvement in English writing, linking ideas, relating results to previous findings, and most importantly providing explanation of each finding that disagree with the existing knowledge or literature.

Since the study design is cross-sectional, the main finding we are looking for is the prevalence of cardiometabolic conditions before and compare it to during the pandemic. However, your findings are not impressive and not reflecting the actual impact of COVID-19 on NCDs burden due to methodology reasons highlighted above. For this, you have to enrich your paper with additional values such as using the analysis of predictors and justifications of such findings.

**Add the following article in your referencing (linking COVID-19 to behavioral risk factors which increase the risk of cardiometabolic conditions)**

[COVID-19 and screen-based sedentary behaviour: Systematic review of digital screen time and metabolic syndrome in adolescents | PLOS ONE](https://journals.plos.org/plosone/article?id=10.1371/journal.pone.0265560)

Available in PubMed also: [COVID-19 and screen-based sedentary behaviour: Systematic review of digital screen time and metabolic syndrome in adolescents - PubMed (nih.gov)](https://pubmed.ncbi.nlm.nih.gov/35312701/)
